# End-tidal carbon dioxide-guided extracorporeal cardiopulmonary resuscitation improves neurological prognosis in patients: a single-center retrospective cohort study

**DOI:** 10.1016/j.bjane.2025.844588

**Published:** 2025-01-23

**Authors:** Xiaozu Liao, Chen Gu, Zhou Cheng, Kepeng Liu, Qing Yin, Binfei Li

**Affiliations:** Zhongshan City People's Hospital, Department of Anesthesiology, Zhongshan, China

**Keywords:** Cardiopulmonary resuscitation, Extracorporeal membrane oxygenation, Prognosis, Risk factors

## Abstract

**Background:**

Extracorporeal Cardiopulmonary Resuscitation (ECPR) is an effective intervention for restoring adequate circulatory perfusion after cardiac arrest. Ensuring high-quality Cardiopulmonary Resuscitation (CPR) before initiating Extracorporeal Membrane Oxygenation (ECMO) is critical to mitigate tissue hypoxia and ischemia. This study aimed to evaluate the effect of End-Tidal Carbon Dioxide (ETCO_2_) Goal-Directed CPR (GDCPR) on neurological function before ECMO using a retrospective case-control analysis.

**Methods:**

The medical records of all patients who received ECPR treated at Zhongshan City People's Hospital were collected between January 2020 and March 2023. In this retrospective cohort study, the patients were divided into Conventional CPR (CCPR) and ETCO_2_-GDCPR groups based on whether ETCO_2_ was used as a guide for CPR.

**Results:**

A total of 71 patients were included, of whom 46 comprised the CCPR group and 25 comprised the GDCPR group. Approximately 37% of patients who received ECPR had good cerebral function at discharge, with a higher rate in the GDCPR group (52%) compared with the CCPR group (28%) (p = 0.047). Multivariate analysis showed that the Highest Interleukin-6 (H-IL6) levels after ECMO (Odds Ratio [OR = 1.001], 95% Confidence Interval [95% CI 1.000–1.003], p = 0.005) was a risk factor for neurological function at discharge. The other risk factors for poor prognosis in patients who received ECPR included pre-ECMO CPR protocols (OR = 10.74, 95% CI 1.90–60.48, p = 0.007) and IL6 levels after ECMO (OR = 1.002, 95% CI 1.001–1.003, p = 0.005). ECMO duration (OR = 0.83, 95% CI 0.74–0.94, p = 0.002) was identified as a protective factor. Patients with short ECMO duration have a poor prognosis. The area under the curve for ECMO duration was 0.86 (0.77–0.94, p < 0.01), while that for H-IL6 was 0.19 (0.09–0.29, p < 0.01).

**Conclusion:**

ETCO_2_-guided ECPR is associated with improved neurological prognosis and patient outcomes. Therefore, monitoring ETCO_2_ levels should be considered a crucial component of evaluating resuscitation efficacy during CPR.

## Introduction

Extracorporeal Cardiopulmonary Resuscitation (ECPR) has gained recognition as a viable treatment for refractory cardiac arrest, with endorsement from multiple clinical guidelines.^1^ ECPR has improved the survival rates and neurological outcomes in patients experiencing cardiac etiology-based cardiac arrest, albeit with survival rates ranging from 10% to 30%.^2^^,^^3^ Improved outcomes are associated with shorter duration of low-flow conditions, elevated arterial blood pH, and reduced serum lactate concentrations in patients who received ECPR.^4^ Therefore, ensuring adequate blood perfusion before initiating Extracorporeal Membrane Oxygenation (ECMO) is crucial.

In clinical practice, chest compressions during CPR are typically assessed using Conventional CPR (CCPR) protocols. Ineffective chest compressions can result from various factors, such as compression location, depth, and other technical aspects that require routine monitoring. These approaches are susceptible to human errors and may vary, particularly when performed by nonspecialized CPR teams.^5^ Consequently, a targeted approach to ensure high-quality CPR delivery before initiating ECMO is urgently required in clinical settings.

End-Tidal Carbon Dioxide (ETCO_2_) levels during CPR correlate with Cardiac Output (CO).^6^ ETCO_2_ is the partial Pressure of Carbon Dioxide (PCO_2_) in exhaled air measured at the end of expiration. CO_2_ is produced by aerobic metabolism in perfused tissues, diffuses from the cells into the blood, and is carried by venous return to the lungs, where it is removed through ventilation. The primary determinants of ETCO_2_ include CO_2_ production, CO, lung perfusion, and alveolar ventilation.^7^ Considering the link between ETCO_2_ and CO, ETCO_2_ could serve as an indirect indicator of cerebral perfusion. Consequently, we propose that ETCO_2_ Goal-Directed CPR (GDCPR) as a precursor to ECMO could provide valuable predictive insights into the neurological status of the patient.

## Materials and methods

This study was approved by the Ethics Committee of the Zhongshan City People's Hospital (Institutional Review Board approval number: K2022-020), and informed consent was obtained from either the patients or their family members.

### Study participants

The study included all patients with cardiac arrest treated at Zhongshan City People's Hospital between January 2020 and March 2023 who received advanced CPR for 20 min without achieving return of spontaneous circulation and subsequently received ECMO support. The inclusion criteria were as follows:

Inclusion criteria and exclusion criteria: Patients aged > 18-years who experienced a witnessed cardiac arrest of cardiac etiology were eligible for inclusion in this study. Patients who exhibited altered consciousness due to other causes, such as trauma or cerebrovascular accidents; were diagnosed with terminal illnesses, including malignant tumors; were aged > 75-years; had irreversible hemorrhagic conditions; experienced cardiac arrest caused by respiratory diseases; or received ECMO support for less than 24h were excluded.

### Research methods

In this study, patients undergoing examination were retrospectively analyzed and categorized into two distinct groups based on the CPR protocol used: CCPR and GDCPR, guided by ETCO_2_ level monitoring. The CCPR group received CPR according to the guidelines outlined by the American Heart Association in 2020. By contrast, the GDCPR group received CPR according to the same American Heart Association guidelines with an additional component of continuous ETCO_2_ monitoring aimed at achieving ETCO_2_ levels exceeding 20 mmHg.^8^ The implementation of EtCO_2_-guided CPR varied among patients due to several factors, primarily due to the availability of CO_2_ monitors in different locations (such as the Intensive Care Unit [ICU] and general floor). Additionally, variations in the healthcare personnel's knowledge and training regarding the application of this method contributed to the decision to use or forego EtCO_2_-guided CPR in certain patients.

### ECMO establishment and management

Venoarterial ECMO (VA-ECMO) was initiated via the femoral artery and venous access at a flow rate of 2.2 L.cm^-2^ × body surface area, maintaining a mean arterial pressure of > 70 mmHg.

The perfusion system consisted of a Medtronic Bio-Pump centrifugal pump, Medtronic oxygenator (Affinity NT), and Carmeda heparin-coated ECMO kit provided by Medtronic, including Medtronic cannulation. Under ultrasound guidance, A 21-Fr cannula was inserted into the femoral vein (insertion depth: 35–45 cm), and A 15-Fr–17-Fr cannula was inserted into the femoral artery (insertion depth: 10–15 cm). After the examination and coronary recanalization, the patient was admitted to the ICU for continued monitoring and treatment. The patients underwent 24h hypothermia treatment (33–34°C) through an ECMO variable temperature tank.

### Data collection

The following demographic and clinical characteristics were obtained from patients: sex, age, weight, medical history, underlying disease, CPR protocol (CCPR or GDCPR), no-flow time, CPR duration, Acute Physiology and Chronic Health Evaluation (APACHE) score, lactate levels before ECMO, lactate levels after ECMO for 24h, ECMO flow on the first day, and Mean Arterial Pressure (MAP) on the first day. Additionally, relevant biochemical indices were assessed before and after ECMO, including the highest Interleukin-6 (H-IL6) level after ECMO (H-IL6), Procalcitonin (PCT) levels, PCT levels after ECMO (H-PCT), C-Reactive Protein (CRP) levels, and CRP levels after ECMO (H-CRP). Furthermore, the post-ECMO Glasgow Coma Scale (GCS) and discharge Cerebral Performance Category (CPC) scores were documented (Table 1).

**Table 1:** Cerebral Performance Category scoring system.

Level	Neurological function
CPC 1	Optimal neurological function: Patient is alert and cognizant, capable of normal life and work activities.
CPC 2	Moderate neurological disability: Patient is alert, capable of part-time work or independent daily activities within a specified environment.
CPC 3	Severe neurological disability: Patient is alert, yet reliant on external assistance for daily activities, retaining limited cognitive function.
CPC 4	Coma and vegetative state: Patient lacks awareness, unconscious of the environment, devoid of cognitive function.
CPC 5	Death: Patient is confirmed as brain-dead or deceased according to conventional criteria.

### Primary endpoint

The primary endpoints of the study were the highest post-ECMO GCS score (assessed daily until discharge) and neurological functional outcomes (CPC scores) at discharge of the CCPR and GDCPR groups. Neurological prognosis was assessed upon discharge using the CPC scale. A CPC score of 1 or 2 was considered indicative of a good neurological prognosis, while a score ranging from 3 to 5 was considered indicative of a poor neurological prognosis.

### Secondary endpoint

The secondary endpoint of the study was the analysis of factors affecting the survival and neurological prognosis of patients who received ECPR at discharge.

### Statistical analyses

Descriptive statistics were calculated for all the variables of interest. Continuous variables were expressed as medians and their corresponding interquartile ranges, while categorical variables were expressed as counts and percentages. For univariate analysis, Fisher's exact test was used for categorical variables, while the Mann-Whitney *U* test was used for continuous variables. The risk factors were initially evaluated using univariate analysis, and significant (p < 0.05) variables were further analyzed using multivariate analysis via multiple logistic regression with forward data elimination. All p-values reported were two-tailed, and statistical significance was set at a p-value of < 0.05. Statistical analyses were conducted using SPSS Statistics for Windows (version 25.0; IBM Corp., Armonk, NY).

## Results

**Figure 1:**
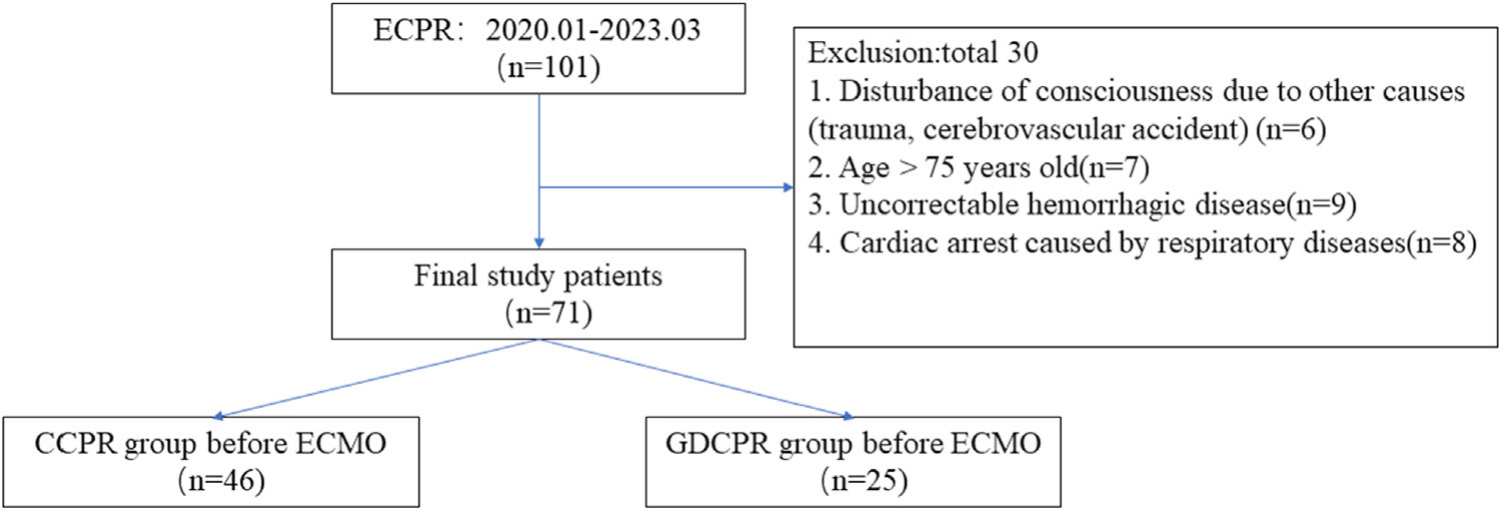
Selection of study patients and study design. CPR, Cardiopulmonary Resuscitation; ECPR, Extracorporeal CPR; CCPR, Conventional CPR; GDCPR, Goal-Directed CPR.

During the study period, 101 patients underwent ECPR, of whom 30 were excluded based on the predefined inclusion criteria, resulting in a final analytical cohort of 71 patients. Among these, the CCPR group comprised 46 patients, while the GDCPR group comprised 25 (Fig. 1).

Table 2 shows the baseline characteristics and clinical outcomes of ECMO in the CCPR and GDCPR groups. The overall survival-to-discharge rate in the ECPR group was 37%. Remarkably, the survival rate in the GDCPR group was 56%, indicating a significant improvement compared with the 30% observed in the CCPR group (p = 0.035). Furthermore, favorable neurological outcomes at discharge were observed in 37% of patients who received ECPR. A significant difference was observed between the GDCPR group, where 52% achieved favorable neurological outcomes, and the CCPR group, where only 28% exhibited favorable neurological outcomes (p = 0.047). Additionally, the GDCPR group consistently displayed higher GCS scores both 24h post-ECMO and throughout the ECMO duration compared with the CCPR group (p < 0.05).

**Table 2:** Comparison of characteristics between the GDCPR group and CCPR group.

Variable	Overall (n = 71)^a^	GDCPR (n = 25)^a^	CCPR (n = 46)^a^	p-value^b^
Sex				0.5
Female	19 (27%)	8 (32%)	11 (24%)	
Male	52 (73%)	17 (68%)	35 (76%)	
Age, years	57 (44–67)	50 (44–60)	58 (45–69)	0.13
Disease				0.2
Myocardial infarct	45 (63%)	12 (48%)	33 (72%)	
Valvular heart disease	11 (15%)	5 (20%)	6 (13%)	
Other primary diseases	15 (21%)	8 (32%)	7 (15%)	
History of disease				
Cerebral infarction				0.4
Yes	7 (9.9%)	1 (4.0%)	6 (13%)	
No	64 (90%)	24 (96%)	40 (87%)	
Hypertension				0.4
Yes	21 (30%)	6 (24%)	15 (33%)	
No	50 (70%)	19 (76%)	31 (67%)	
Diabetes				0.2
Yes	15 (21%)	3 (12%)	12 (26%)	
No	56 (79%)	22 (88%)	34 (74%)	
Before ECMO				
No-flow time, min	1.46 (1)	1.64 (1–3)	1.36 (1–3)	0.13
CPR duration, min	34 (29–55)	45 (30–60)	31 (29–50)	0.3
APACHE score	30 (25–37)	29 (20–31)	33 (27–38)	0.008
Lactate level, mmoL.L^-1^ before ECMO	13.9 (10.3–15.0)	13.7 (11.0–15.0)	14.9 (9.7–15.0)	0.7
After ECMO				
ECMO flow, L.min^-1^ on the first day	2.98 (2.58–3.26)	2.80 (2.50–3.00)	3.00 (2.71–3.44)	0.2
MAP, mmHg on the first day	75 (65–80)	76 (70–80)	70 (57–78)	0.056
Lactate level, mmoL.L^-1^ 24h after ECMO	5.80 (2.70–12.60)	2.3 (1.75–7.05)	8.9 (3.90–15)	0.00
IABP				0.6
Yes	20 (28%)	8 (32%)	12 (26%)	
No	51 (72%)	17 (68%)	34 (74%)	
Infect				0.7
Yes	52 (73%)	19 (76%)	33 (72%)	
No	19 (27%)	6 (24%)	13 (28%)	
H-IL6, pg.mL^-1^	865 (324–2,685)	1,214 (323–2,724)	668 (355–2,464)	0.6
CRP, mg.L^-1^ 24h after ECMO	56 (14–107)	76 (22–125)	53 (12–84)	0.4
H-CRP, mg.L^-1^	130 (76–236)	130 (99–241)	130 (75–236)	0.5
PCT, ng.mL^-1^	19 (7–40)	16 (7–30)	23 (7–48)	0.3
GCS 24h after ECMO	8 (4–13)	10 (8–13)	6 (4–13)	0.063
H-GCS	10 (4–15)	13 (10–15)	8 (4–15)	0.040
Duration of ECMO, days	2.80 (1.20–4.83)	3.20 (2.25–6.83)	1.95 (1.00–4.14)	0.004
ECMO weaning				0.071
Yes	38 (54%)	17 (68%)	21 (46%)	
No	33 (46%)	8 (32%)	25 (54%)	
Length of hospital stay, days	17 (5–30)	21 (14–35)	15 (3–23)	0.093
CPC score at discharge				0.047
Brain function is good	26 (37%)	13 (52%)	13 (28%)	
Brain dysfunction	45 (63%)	12 (48%)	33 (72%)	
Survival to discharge				0.035
Yes	28 (39%)	14 (56%)	14 (30%)	
No	43 (61%)	11 (44%)	32 (70%)	

a n (%), median (IQR).

b Pearson's Chi-Square test; Wilcoxon rank-sum test; Fisher's exact test. CPR, Cardiopulmonary Resuscitation; CCPR, Conventional CPR; GDCPR, Goal-Directed CPR; IABP, Intra-Aortic Balloon Pump; CPC, Cerebral Performance Category; CRP, C-Reactive Protein; GCS, Glasgow Coma Scale; H-GCS, Highest GCS after ECMO; H-IL6, Highest IL6 after ECMO; PCT, Procalcitonin; H-PCT, Highest PCT after ECMO; H-CRP, highest CRP after ECMO.

Univariate analysis (Table 3) revealed that several factors significantly influenced patient prognosis, including patient age, pre-ECMO APACHE score, lactate levels, CPR protocol, 24h post-ECMO lactate levels, post-ECMO H-IL6 levels, concurrent intra-aortic balloon pump support, ECMO duration, ICU stay, and overall hospital stay (p < 0.05).

**Table 3:** Univariate analysis of the prognosis at hospital discharge of patients who received ECPR.

Variable	Overall (n = 71)^a^	Survivor (n = 28)^a^	Non-survivor (n = 43)^a^	p-value^b^
Sex				0.4
Female	19 (27%)	9 (32.1%)	10 (23.3%)	
Male	52 (73%)	19 (67.9%)	33 (76.7%)	
Age	57 (44–67)	49 (43–59)	61 (44–70)	0.04
Disease				0.23
Myocardial infarct	45 (63%)	17 (60.7%)	28 (65.1%)	
Valvular heart disease	12 (17%)	3 (10.7%)	9 (20.9%)	
Fulminant myocarditis	7 (10%)	5 (17.9%)	2 (4.7%)	
Other primary diseases	7 (10%)	3 (10.8%)	4 (9.3%)	
CPR protocol				0.035
GDCPR	25 (35.2%)	14 (50%)	11 (25.6%)	
CCPR	46 (64.8%)	14 (50%)	32 (74.4%)	
IABP				0.03
Yes	18 (25%)	11 (39.3%)	7 (16.3%)	
No	53 (75%)	17 (60.7%)	36 (83.7%)	
Infarction				0.84
Yes	7 (9.9%)	3 (10.7%)	4 (9.3%)	
No	64 (90%)	25 (89.3%)	39 (90.7%)	
Hypertension				0.38
Yes	22 (31%)	7 (25%)	15 (34.9%)	
No	49 (69%)	21 (75%)	28 (65.1%)	
Diabetes				0.45
Yes	16 (23%)	5 (17.9%)	11 (25.6%)	
No	55 (77%)	23 (82.1%)	32 (74.4%)	
No-flow time, min	1.46 (1–3)	1.46 (1–3)	1.46 (1–3)	0.86
CPR duration, min	34 (28–55)	30 (20–53)	35 (30–55)	0.07
APACHE score	31 (25–37)	26 (21–32)	32 (28–38)	0.001
Duration of ECMO, h	67.2 (28.0–114.24)	90 (52.5–121.8)	48 (24–85.4)	0.014
Length of ICU stay, days	7 (2–12.57)	12.23 (8.86–19.88)	3 (1.4–8.0)	0.00
Length of hospital stay, days	16 (4–31)	33 (17–54.5)	9 (2–17)	0.00
Lactate level, mmoL.L^-1^ before ECMO	13.9 (10.0–15.0)	11.35 (9.71–14.7)	15.0 (10.9–15.0)	0.004
Lactate level, mmoL.L^-1^ 24h after ECMO	5.8 (2.7–12.6)	4.0 (1.85–6.25)	10.0 (3.9–15.0)	0.001
H-IL6, pg.mL^-1^	736 (323–2,500)	323 (136–735.5)	1805 (567–5,000)	0.00
ECMO flow, L.min^-1^ on the first day	2.98 (2.55–3.22)	2.85 (2.41–3.00)	3.00 (2.78–3.40)	0.033
MAP, mmHg on the first day	75 (65–80)	78 (71–85)	70 (56–78)	0.001
Infect				0.78
Yes	52 (73%)	20 (71.4%)	32 (74.4%)	
No	19 (27%)	8 (28.6%)	11 (25.6%)	
H-PCT, ng.mL^-1^	19 (6.26–39.52)	16.35 (4.56–21.63)	25 (7.1–47.24)	0.077

a n (%), median (IQR).

b Pearson's Chi-Square test; Wilcoxon rank-sum test; Fisher's exact test. CPR, Cardiopulmonary Resuscitation; CCPR, Conventional CPR; GDCPR, Goal-Directed CPR; H-IL6, Highest IL6 after ECMO; PCT, Procalcitonin; H-PCT, Highest PCT level after ECMO oxygenation.

Multivariate analysis showed that the H-IL6 level after ECMO (Odds Ratio [OR = 1.001], 95% Confidence Interval [95% CI 1.000–1.003], p = 0.005) was a risk factor for neurological outcome at discharge (Additional Tables 1 and 2). Furthermore, multivariate analysis revealed that the CPR protocol (OR = 10.74, 95% CI 1.90–60.48, p = 0.007) and post-ECMO H-IL6 levels (OR = 1.002, 95% CI 1.001–1.003, p = 0.005) were associated with survival in patients who received ECPR, indicating that ETCO_2_-GDCPR is linked to a better prognosis. Conversely, ECMO duration (OR = 0.83, 95% CI 0.74–0.94, p = 0.002) emerged as a protective factor that positively influenced survival (Table 4), suggesting that a shorter ECMO duration is correlated with a poor prognosis.

**Table 4:** Multivariate logistic regression analysis of prognostic factors of survival at hospital discharge.

	OR (95% CI)	p-value
**CPR Protocol**	10.74 (1.90–60.48)	0.007
**Length of ICU stay**	0.83 (0.74–0.94)	0.002
**H-IL6**	1.002 (1.001–1.003)	0.005

OR, Odds Ratio; CI, Confidence Interval.

**Figure 2:**
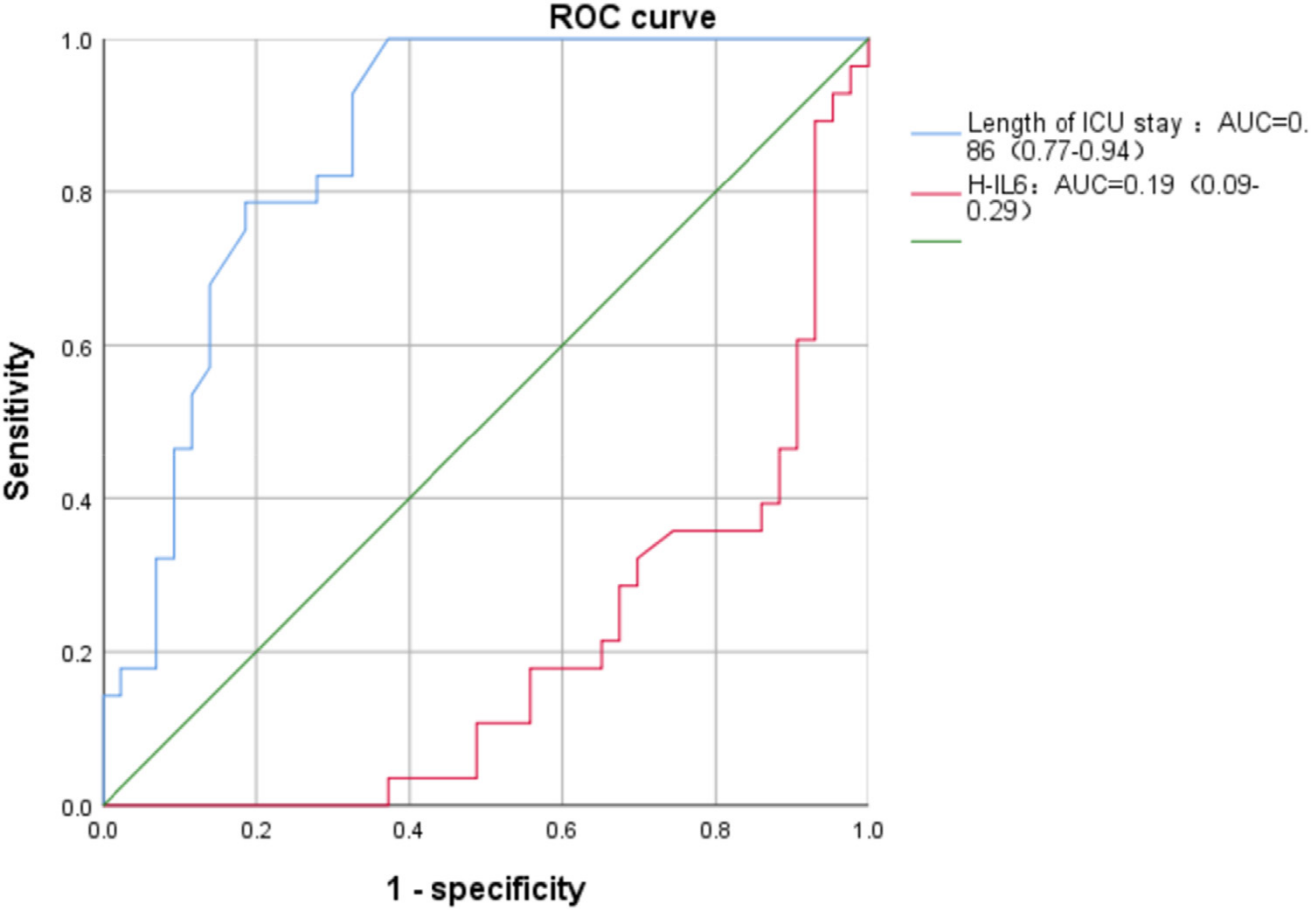
ROC curves for ICU length of stay and H-IL6 levels.

Figure 2 illustrates the Receiver Operating Characteristic (ROC) curve analysis of ECMO duration and H-IL6 level. Both ECMO duration (Area Under the Curve [AUC] = 0.86 [0.77–0.94], p < 0.01) and H-IL6 level (AUC = 0.19 [0.09–0.29], p < 0.01) were significant indicators.

## Discussion

The findings of this investigation suggest that ETCO_2_-guided ECPR offers significant benefits in neurological outcomes and survival-to-discharge rates compared with conventional CCPR. Therefore, ETCO_2_-guided ECPR is a robust and independent predictor of patient prognosis.

Previous studies have demonstrated that when CCPR fails to restore effective circulatory perfusion, ECPR can rapidly reinstate circulation, thereby enhancing the chances of survival and favorable neurological outcomes.^9^ Prolonged periods of inadequate perfusion during conventional resuscitation before ECPR initiation have been linked to less favorable clinical outcomes.^4^ In China, public hospitals are crucial in providing emergency and critical care services to patients with severe conditions, including those requiring VA-ECMO. Although VA-ECMO is available, the costs associated with this treatment are primarily covered by national healthcare insurance across public, private, and university-affiliated hospitals. In China, ECMO is categorized as a restricted technology, with strict criteria for patient selection to enhance its value and efficacy. Hence, delivering high-quality CPR before initiating ECMO is essential.

The study revealed that patients who received ETCO_2_-GDCPR exhibited higher scores in key parameters, including the 24h ECMO GCS, best GCS during ECMO, and CPC score at discharge, compared with their counterparts who received traditional CCPR.^10^^,^^11^ Previous studies have identified multiple factors that can influence neurological outcomes in patients undergoing ECPR, including no-flow time, compression duration and quality, and ECMO initiation time.^12^, ^13^, ^14^ Our findings revealed that patients who received CPR without ETCO_2_ monitoring had longer compression times than the control group but achieved better neurological outcomes. This observation further underscores the significance of effective compression during resuscitation. This discrepancy may be attributed to the absence of standardized quality control in CPR practices, making it challenging to ensure effective CPR. Multiple variables influence CPR quality, including skill level and training history of CPR operators.^15^ ETCO_2_ is a noninvasive measure commonly used by many emergency medical services for CPR quality monitoring; however, it has not yet been widely adopted in China.^16^^,^^17^ Numerous studies have corroborated the effectiveness of ETCO_2_ in evaluating CO following pre-hospital cardiac arrest.^18^

Furthermore, our results showed that H-IL6 level after ECMO (OR = 1.002, 95% CI 1.001–1.003, p = 0.005) was a risk factor for neurological outcome and prognosis at discharge. The AUC for H-IL6 level was 0.19 (0.09–0.29, p < 0.01). Multiple factors influence the neurological outcomes in patients receiving ECPR. Severe cardiopulmonary failure preceding ECMO treatment increases susceptibility to hypoxic-ischemic injury.^19^^,^^20^ Previous studies have substantiated the association among elevated systemic inflammation marker levels, endothelial activation, fibrinolysis indicators, mortality, and aberrant neurological function in ECMO-supported patients.^21^ Our findings align with this observation, indicating that severe inflammatory reactions (H-IL6) associated with ECMO are risk factors for neurological outcomes at the time of hospital discharge. Different compression strategies did not independently affect neurological function, highlighting the multifactorial nature of these outcomes. Previous studies have demonstrated that excessive inflammatory responses can adversely affect brain function, reinforcing the need for effective monitoring and management after cardiac arrest.^22^ IL6 may serve as a potential indicator of ischemic brain injury severity and its clinical implications.^23^^,^^24^ In this study, the ROC curve analyses were conducted to explore the role of IL6 in predicting important clinical outcomes. The AUC for IL6 was only 0.19. This finding aligns with our understanding of the complexities involved in patients with ECMO support, who often present with multiple comorbidities and complications, making it challenging to provide an accurate prognostic assessment using only a single biomarker. During ECMO, the levels of IL6, an inflammatory marker, can be influenced by several factors, including infection, tissue injury, and the patient's baseline health status. Therefore, the diagnostic efficacy of IL6 may be limited in this complex context. Although IL6 was not an effective independent prognostic indicator in this study, this result does not negate its potential clinical significance.

The multivariate analysis in this study identified the ECPR protocol as an independent risk factor influencing patient prognosis, with ETCO_2_-guided ECPR associated with a more favorable outcome. This observation aligns with the findings of previous research, which linked better post-ECPR outcomes to shorter low-flow durations, elevated pH levels, and lower lactate concentrations.^25^ Elevated ETCO_2_ levels benefit the vital organs and reflect CO during CPR.^26^ Consequently, ETCO_2_-GDCPR enabled precise chest compressions, ensured efficient circulatory perfusion before ECMO initiation, and reduced systemic tissue hypoxia. Animal studies have demonstrated that reduced ETCO_2_ levels correlate with decreased carotid artery blood pressure and flow during CPR and are associated with cerebral and renal injury severity.^27^

Additionally, multivariate analysis underscored the duration of ICU stay as a protective factor influencing the prognosis of patients who received ECPR. Patients who survived until discharge had longer ICU stays than non-survivors, which is consistent with the findings of Yeh et al.^28^ Contrary to previous studies suggesting that an extended ICU stay may adversely affect patient outcomes, this study included patients who received ECPR with clinical conditions more severe than those primarily experiencing cardiogenic shock.^29^ In this study, the non-surviving group had an ICU stay of only 3-days, likely due to suboptimal CPR quality and subsequent clinical deterioration. Additionally, the levels of perfusion and illness severity markers (higher lactate levels) were higher in the surviving group than in the non-surviving group. This finding underscores the importance of high-quality CPR as a critical factor in improving outcomes, which is consistent with the results of a study conducted by Li et al.^30^ CPR quality significantly impacts tissue and organ perfusion, with a notable proportion (74.4%) of patients who experienced cardiac arrest in the non-surviving group undergoing CCPR without quality monitoring.^31^^,^^32^ Previous research has demonstrated that the effectiveness of CPR significantly affects patient prognosis.^33^ The incidence of infection among patients who received ECPR is 73%, potentially initiating a systemic inflammatory response, cytokine release, and distant organ injury, thereby contributing to systemic shock. Infections tend to prolong ICU stays and increase mortality rates.^34^

However, this study has certain limitations. First, our study was a retrospective analysis conducted at a single center. The small sample size limits the reliability of our results, and the retrospective design introduces biases, such as incomplete records and the absence of randomization. Our preliminary findings suggest that ETCO_2_-guided CPR may have clinical value. Specifically, early findings indicated that ETCO_2_ levels may correlate with neurological outcomes in patients experiencing cardiac arrest on ECMO. To further explore this, we reviewed the historical data of the patients. Second, the study excluded patients with ECMO support duration of less than 24h and those with uncontrolled bleeding before ECMO initiation, which may introduce bias due to the limited sample size. Third, variability in the treatment approaches used by the attending physicians could have affected the study outcomes. Therefore, we recommend conducting further multicenter, large-scale, independent cohort studies to validate these findings, as previous studies have indicated that patients with myocardial infarction have lower survival rates following CPR than those with myocarditis undergoing ECMO. This suggests that the presence of such comorbidities could influence patient outcomes, although our findings were not significant. Variability in patient prognosis is influenced by multiple factors.^35^ Additionally, the absence of controls for CPR quality-related variables affected the robustness of the results. We emphasize the need for future studies with larger sample sizes. Conducting matched analyses could provide more reliable results and help elucidate the impact of underlying health conditions on patient outcomes.

## Conclusion

Using an ETCO_2_-guided compression strategy during ECPR is associated with enhanced neurological prognostic markers, thereby positively influencing patient outcomes. Consequently, the assessment of ETCO_2_ during CPR is a significant and relevant parameter for evaluating the effectiveness of resuscitative interventions.

## Conflicts of interest

The authors declare no have conflicts of interest.

## Appendix Supplementary materials

Image, application 1
