# Supplementary material for: End-tidal carbon dioxide-guided extracorporeal cardiopulmonary resuscitation improves neurological prognosis in patients: a single-center retrospective cohort study
Source: Braz J Anesthesiol. 2025 Jan 23;75(3):844588. doi: 10.1016/j.bjane.2025.844588 (PMC11849071; doi:10.1016/j.bjane.2025.844588)
Supplement: Supplementary file 1 [file mmc1.docx]

**BJAN-D-24-00377_Supplementary Material**

**Additional Table 1** Univariate analysis of the neurological prognosis at discharge of patients who received ECPR.

| **Variable** | **Overall**  **(n = 71)^a^** | **Good neurological prognosis**  **(n = 26)^a^** | **Poor neurological prognosis**  **(n = 45)^a^** | **p-value^b^** |
| --- | --- | --- | --- | --- |
| Sex |  |  |  | 0.4 |
| Female | 19 (27%) | 9 (34.6%) | 10 (22.2%) |  |
| Male | 52 (73%) | 17 (66.4%) | 35(77.8%) |  |
| Age | 55 (44–67) | 51 (42–61) | 60 (44–69) | 0.07 |
| Disease |  |  |  | 0.23 |
| Myocardial infarct | 45 (63%) | 15 (57.7%) | 30 (66.7%) |  |
| Valvular heart disease | 12 (17%) | 3 (11.5%) | 9 (20%) |  |
| Fulminant myocarditis | 7 (10%) | 5 (19.3%) | 2 (4.4%) |  |
| Other primary diseases | 7 (10%) | 3 (11.5%) | 4 (8.9%) |  |
| CPR Protocol |  |  |  | 0.047 |
| GDCPR | 25 (35.2%) | 13 (50%) | 12 (26.7%) |  |
| CCPR | 46 (64.8%) | 13 (50%) | 33 (73.3%) |  |
| Infarction |  |  |  | 0.642 |
| Yes | 7 (9.9%) | 2 (7.6%) | 5 (11.1%) |  |
| No | 64 (90%) | 24 (92.4%) | 40 (88.9%) |  |
| Hypertension |  |  |  | 0.574 |
| Yes | 22 (31%) | 7 (28%) | 15 (33.3%) |  |
| No | 49 (69%) | 19 (72%) | 30 (66.7%) |  |
| Diabetes |  |  |  | 0.612 |
| Yes | 16 (23%) | 5 (19.2%) | 11 (24.4%) |  |
| No | 55 (77%) | 21 (80.8%) | 34 (75.6%) |  |
| No-flow time, min | 1.46 (1–3) | 1.46 (1–3) | 1.47 (1–3) | 0.802 |
| CPR duration, min | 34 (28–55) | 29 (20– 56) | 35 (30–52) | 0.057 |
| APACHE score | 31 (25– 37) | 27 (20–33) | 32 (28–38) | 0.005 |
| Lactate level, mmoL.L^-1^ before ECMO | 13.9 (10.0–15.0) | 11.35 (9.6–14.1) | 15.0 (10.4–15.0) | 0.004 |
| Lactate level, mmoL.L^-1^ 24h after ECMO | 5.8 (2.7–12.6) | 3.6 (1.75–6.45) | 10.0 (3.9–15.0) | 0.001 |
| H-IL6, pg.mL^-1^ | 736 (323–2,500) | 323 (123–634) | 1,805 (583–5,000) | 0.00 |
| ECMO flow, L.min^-1^ on the first day | 2.98 (2.55–3.22) | 2.9 (2.4–3.0) | 3.00 (2.6–3.4) | 0.12 |
| MAP, mmHg on the first day | 75 (65–80) | 78 (75–86) | 67 (56–78) | 0.00 |
| Infect |  |  |  | 0.98 |
| Yes | 52 (73%) | 19 (73.1%) | 33 (71.7%) |  |
| No | 19 (27%) | 7 (26.9%) | 12 (28.3%) |  |
| H-PCT, ng.mL^-1^ | 19 (6.26–39.52) | 15.6 (4.4–18.9) | 25 (7.6–46.4) | 0.034 |

^a^ n (%), median (IQR).

^b^ Pearson’s Chi-Square test; Wilcoxon rank-sum test; Fisher’s exact test

CPR, Cardiopulmonary Resuscitation; CCPR, Conventional CPR; GDCPR, Goal-Directed CPR; H-IL6, Highest IL6 after ECMO; PCT, Procalcitonin; H-PCT, Highest PCT level after ECMO oxygenation.

**Additional Table 2** Multivariate logistic regression analysis of neurological prognosis at hospital discharge of patients who received ECPR.

|  | **OR (95% CI)** | **p-value** |
| --- | --- | --- |
| **H-IL6** | 1.001 (1.00–1.003) | 0.005 |

OR, Odds Ratio, CI, Confidence Interval.
